# Primary omental smooth muscle tumor in an adult male: a diagnostic dilemma for leiomyoma: a case report

**DOI:** 10.1186/s13256-024-04537-9

**Published:** 2024-05-05

**Authors:** Yukari Ono, Yoichiro Okubo, Kota Washimi, Yo Mikayama, Tsunehiro Doiuch, Chie Hasegawa, Emi Yoshioka, Kyoko Ono, Manabu Shiozawa, Tomoyuki Yokose

**Affiliations:** 1 Department of Pathology, Kanagawa Cancer Center, 2-3-2, Nakao, Asahi-Ku, Yokohama, Kanagawa 241-8515 Japan; 2 Department of Gastrointestinal Surgery, Kanagawa Cancer Center, 2-3-2, Nakao, Asahi-Ku, Yokohama, Kanagawa 241-8515 Japan; 3 Department of Diagnostic and Interventional Radiology, Kanagawa Cancer Center, 2-3-2, Nakao, Asahi-Ku, Yokohama, Kanagawa 241-8515 Japan

**Keywords:** Omentum, Leiomyoma, Smooth muscle tumor, Ki-67 labeling index, Case report

## Abstract

**Background:**

The greater omentum comprises peritoneal, adipose, vascular, and lymphoid tissues. Most omental malignancies are metastatic tumors, and the incidence of primary tumors is rare. We report on a prior omental smooth muscle tumor case in an adult male patient.

**Case presentation:**

A 54-year-old Japanese male patient with no relevant medical history was diagnosed with an abdominal mass during a routine medical checkup. Subsequent contrast-enhanced computed tomography revealed a mass of approximately 3 cm in size in the greater omentum, and a laparotomy was performed. A 27 × 25 × 20 mm raised lesion was found in the omentum. Microscopically, spindle cells were observed and arranged in whorls and fascicles. Individual tumor cells had short spindle-shaped nuclei with slightly increased chromatin and were characterized by a slightly eosinophilic, spindle-shaped cytoplasm. The mitotic count was less than 1 per 50 high-power fields. The tumor cells showed positive immunoreactivity for α smooth muscle actin, HHF35, and desmin on immunohistochemical examination. The Ki-67 labeling index using the average method was 1.76% (261/14806). No immunoreactivity was observed for any of the other tested markers. We considered leiomyoma owing to a lack of malignant findings. However, primary omental leiomyoma has rarely been reported, and it can be difficult to completely rule out the malignant potential of smooth muscle tumors in soft tissues. Our patient was decisively diagnosed with a primary omental smooth muscle tumor considering leiomyoma. Consequently, the patient did not undergo additional adjuvant therapy and was followed up. The patient was satisfied with treatment and showed neither recurrence nor metastasis at the 13-month postoperative follow-up.

**Discussion and conclusion:**

We encountered a primary smooth muscle tumor of the greater omentum with no histological findings suggestive of malignancy in an adult male patient. However, omental smooth muscle tumors are extremely difficult to define as benign, requiring careful diagnosis. Further case reports with long-term follow-up and case series are required to determine whether a true omental benign smooth muscle tumor (leiomyoma) exists. In addition, proper interpretation of the Ki-67 labeling index should be established. This case study is a foundation for future research.

## Background

The greater omentum is a two-layered membrane that arises from the greater curvature of the stomach, extends down to cover the abdominal organs, and folds back to join the transverse colon [1]. This organ mainly comprises the peritoneal and adipose tissues and includes vessels and lymphoid tissue [2, 3]. The greater omentum contains omentum-associated lymphoid tissues (OALTs), also called “milky spots” [2]. OALT promptly filters lymphocytes, including various types of cells, and is responsible for the immune defense in the abdominal cavity [4]. Notably, OALT has a significant impact on peritoneal carcinomatosis because it is also responsible for tumor cell filtration [4–6]. Therefore, most malignancies of the greater omentum are metastatic, and the incidence of primary tumors is rare [1, 7]. Herein, we report a case of a primary smooth muscle tumor arising in the greater omentum in an adult male, along with its histological characteristics. The tumor was challenging to definitively diagnose as leiomyoma.

## Case presentation

The patient was a 54-year-old Japanese male with no medical treatment history. However, an abdominal ultrasound performed during a routine medical checkup incidentally detected a solid mass in the abdominal cavity. The patient was referred to the medium-scale hospital for a more detailed examination, and contrast-enhanced computed tomography (CT) was performed. The results showed an abdominal mass approximately 3 cm in size in the greater omentum, near the posterior wall of the stomach and transverse colon. Subsequently, the patient was referred to the department of gastrointestinal surgery at our hospital for further examination to obtain a definitive diagnosis.

Results of a detailed examination at our hospital revealed that the patient, working as an office employee, had no significant medical or surgical history. He smoked 20 cigarettes daily since age 20 years and consumed 250 mL of beer daily. The family history was significant for cancer, with his father diagnosed with rectal cancer at age 65 years, his mother with breast cancer at 60 years, and his maternal grandmother with pancreatic cancer at 55 years. Upon physical examination, the patient was asymptomatic with no abnormal physical or neurological findings. Vital signs were within normal limits. Laboratory investigations revealed a normal complete blood count, with white blood cells at 7000/μL, red blood cells at 4.69 million/μL, platelets at 28.1 × 10^3^/μL, and hemoglobin at 15.8 g/dL. Renal function tests were within normal limits, with creatinine at 0.77 mg/dL and blood urea nitrogen at 11 mg/dL. Electrolyte levels were stable, with sodium (Na) at 141 mmol/L, chloride (Cl) at 105 mmol/L, potassium (K) at 4.3 mmol/L, and calcium (Ca) at 9.5 mg/dL. Liver enzymes, including aspartate aminotransferase at 19 U/L, alanine aminotransferase at 18 U/L, gamma-glutamyl transferase at 61 U/L, albumin at 4.5 g/dL, total protein at 7 g/dL, and total bilirubin at 1 mg/dL, were within normal ranges. Inflammatory markers were low, with C-reactive protein at 0.06 mg/dL; hemoglobin A1c was 5.3%, indicating no evidence of diabetes. Regarding the patient’s medication history, there were no medications being taken prior to the diagnosis, and notably, the patient had not undergone any form of chemotherapy before or after surgery. No medications were administered prior to diagnosis, with attention focused on diagnostic assessments and surgical intervention for the primary omental smooth muscle tumor. The patient’s history did not suggest any environmental or occupational exposures contributing to his condition. The case was meticulously documented, considering the detailed family history of cancer and the patient’s lifestyle habits, such as smoking and alcohol consumption, to provide a comprehensive background for diagnosis and management.

**Fig. 1:**
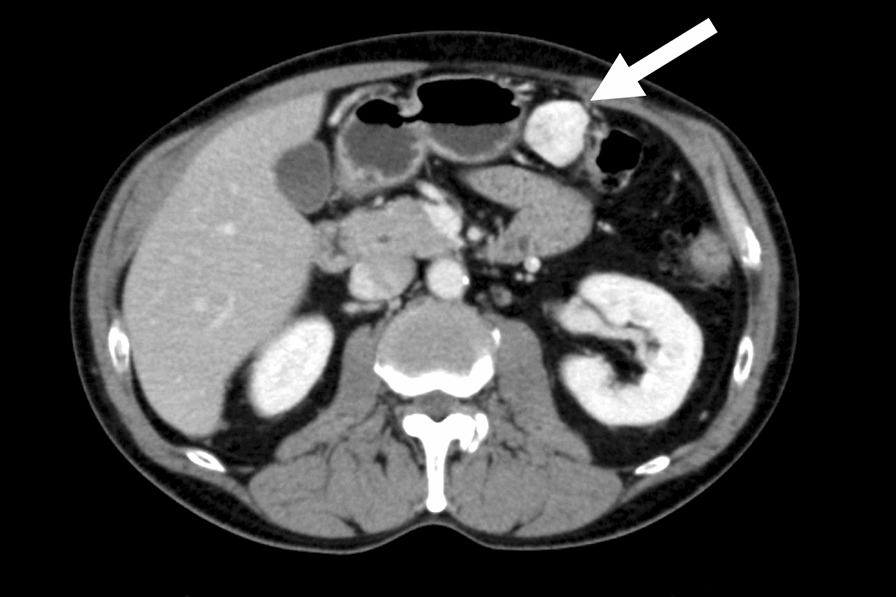
Representative contrast-enhanced computed tomography images in this case. A contrast-enhanced computed tomography scan showing an abdominal mass approximately 3 cm in size in the greater omentum (arrow indicates tumor)

The patient had no specific symptoms, such as abdominal pain, and no ascites or other lesions were detected on whole body evaluation. Contrast-enhanced CT at our hospital also demonstrated a 3 cm in size solitary mass in the omentum (Fig. 1). Open laparotomy was eventually performed because malignancy could not be ruled out clinically, and a needle biopsy could have caused tumor dissemination. The intraoperative findings were a solitary tumor within the omentum, with no evidence of adhesion to the adjacent posterior wall of the stomach or transverse colon.

**Fig. 2:**
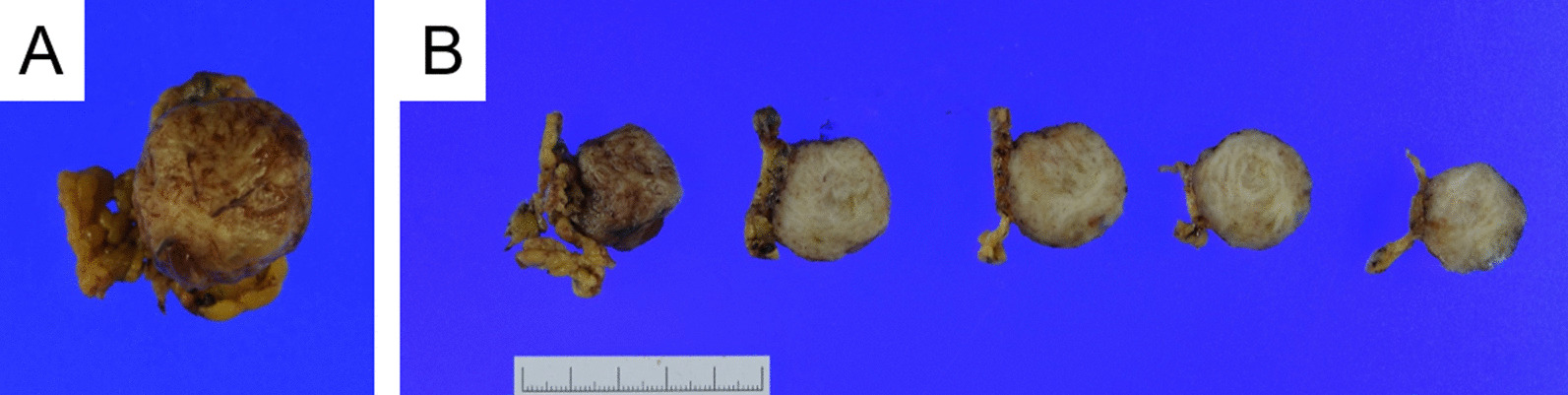
Macroscopic findings of the tumor. **A** Formalin fixation showing that macroscopically, the specimen is a 27 mm × 25 mm × 20 mm elevated lesion with slight adipose tissue in the periphery. The surface is somewhat rough, but no obvious capsular rupture or tumor exposure is observed. **B** A solid tumor, which is grayish-white and heterogeneous, is observed on the cut surface. Although the general appearance is elastic and slightly firm, no obvious calcification or necrosis is observed

**Fig. 3:**
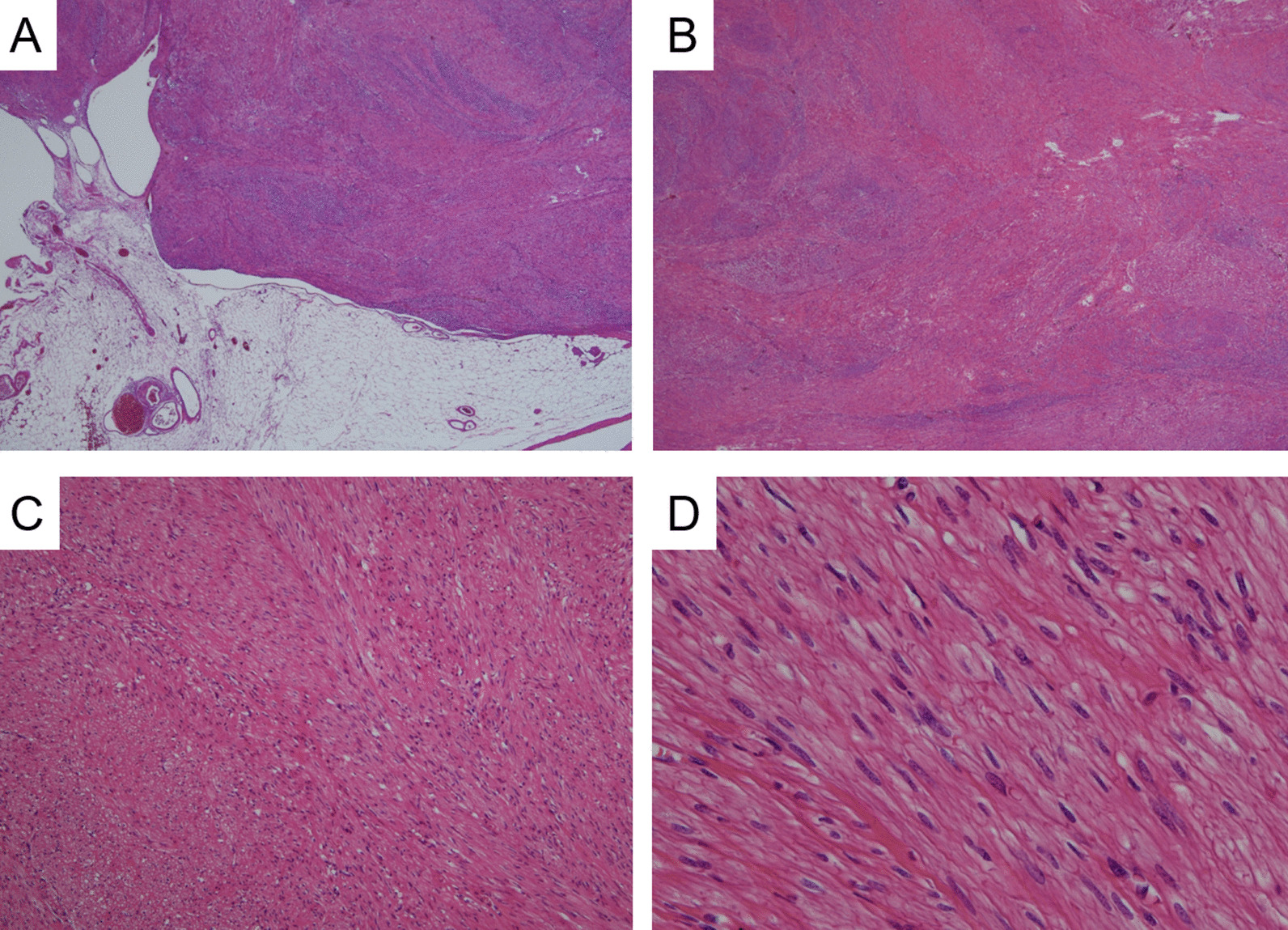
Microscopic findings at various magnifications in this tumor. **A**, **B** Low-power-field view. Tumor cells are arranged in whorls and fascicles. Hyalinization or necrosis is not observed. In addition, adipose tissue is adherent to the periphery, but no obvious irregularities are detected (hematoxylin and eosin staining, magnification ×20). **C** Medium-power field view of the case. The tumor cells arranged in the whorls and fascicles are clarified. Abundant blood vessels suggestive of angioleiomyoma are not observed. In addition, neither nuclear atypia nor mitotic figures, recognizable at this magnification, could be detected (hematoxylin and eosin staining, magnification ×100). **D** High-power field view of the patient. Individual tumor cells had short spindle-shaped nuclei with a slight increase in chromatin and slightly eosinophilic, spindle-shaped cytoplasm (hematoxylin and eosin staining, magnification ×400)

Macroscopically, the specimen after formalin fixation was a 27 mm × 25 mm × 20 mm elevated lesion with slight adipose tissue in the periphery. Solid tumors with heterogeneous grayish-white cut surfaces were observed, and no obvious calcification or necrosis was observed (Fig. 2). Microscopically, spindle cells were observed and arranged in whorls and fascicles. Individual tumor cells had short spindle-shaped nuclei with a slight increase in chromatin, and a slightly eosinophilic, spindle-shaped cytoplasm was observed. No hyalinization, calcification, or tumor necrosis was observed in the background. The mitotic count was less than 1 per 50 high-power fields. Furthermore, no abundance of blood vessels were observed to suggest angioleiomyoma (Fig. 3).

**Fig. 4:**
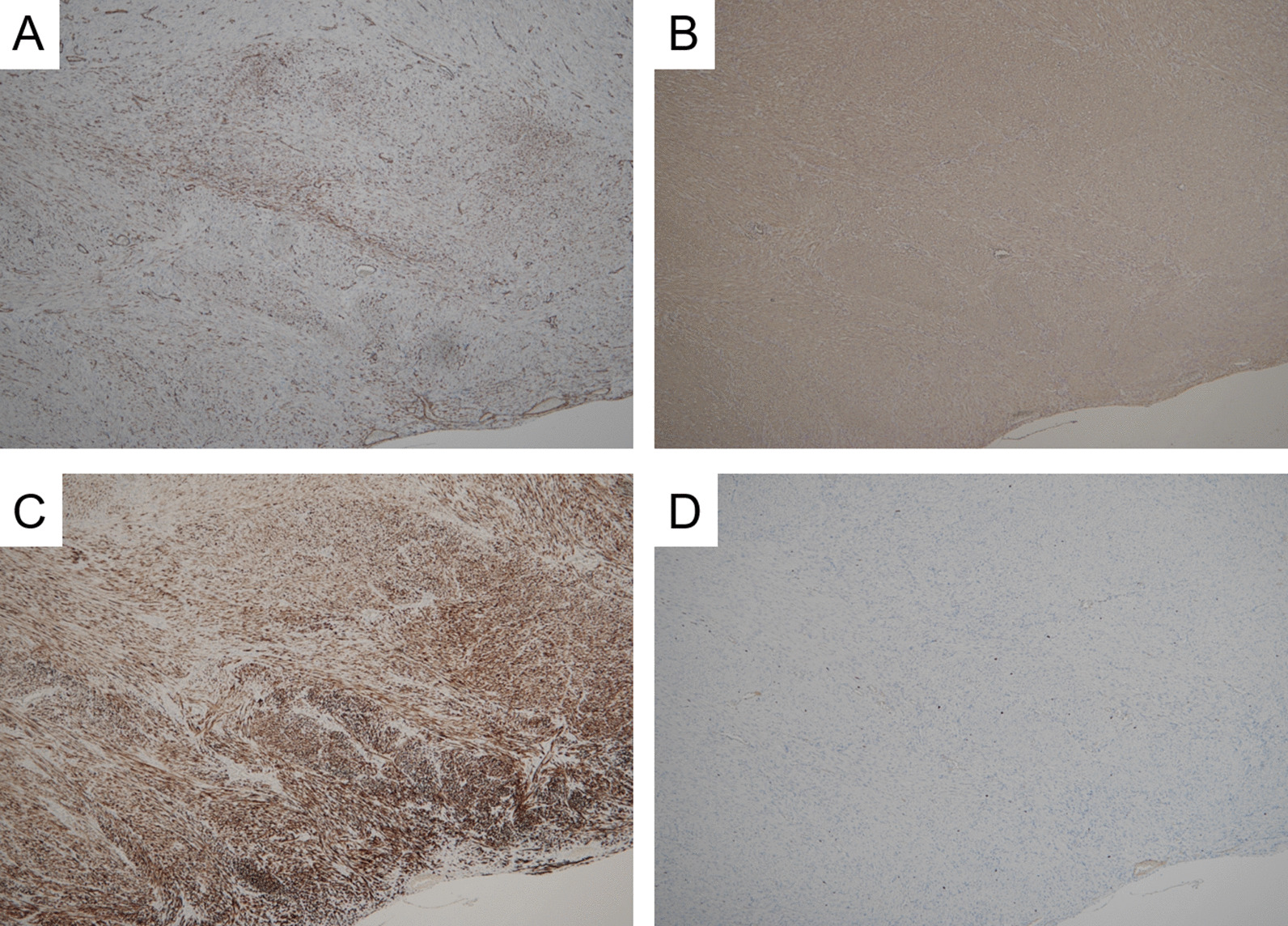
Representative images showing immunoreactivity in the tumor. **A**–**C** On immunohistochemical examination, tumor cells show positive immunoreactivity focally to αSMA (**A**) and diffusely to HHF 35 and desmin (**C** and **D**, respectively). **D** Only a few Ki-67 positive cells are observed. According to the image analysis software, the Ki-67 labeling index using the average method is 1.76% (261/14806), while it is 3.51% (43/1226) using the hotspot method (**A** αSMA, **B** HHF35, **C** desmin, **D** Ki-67, ×100)

**Fig. 5:**
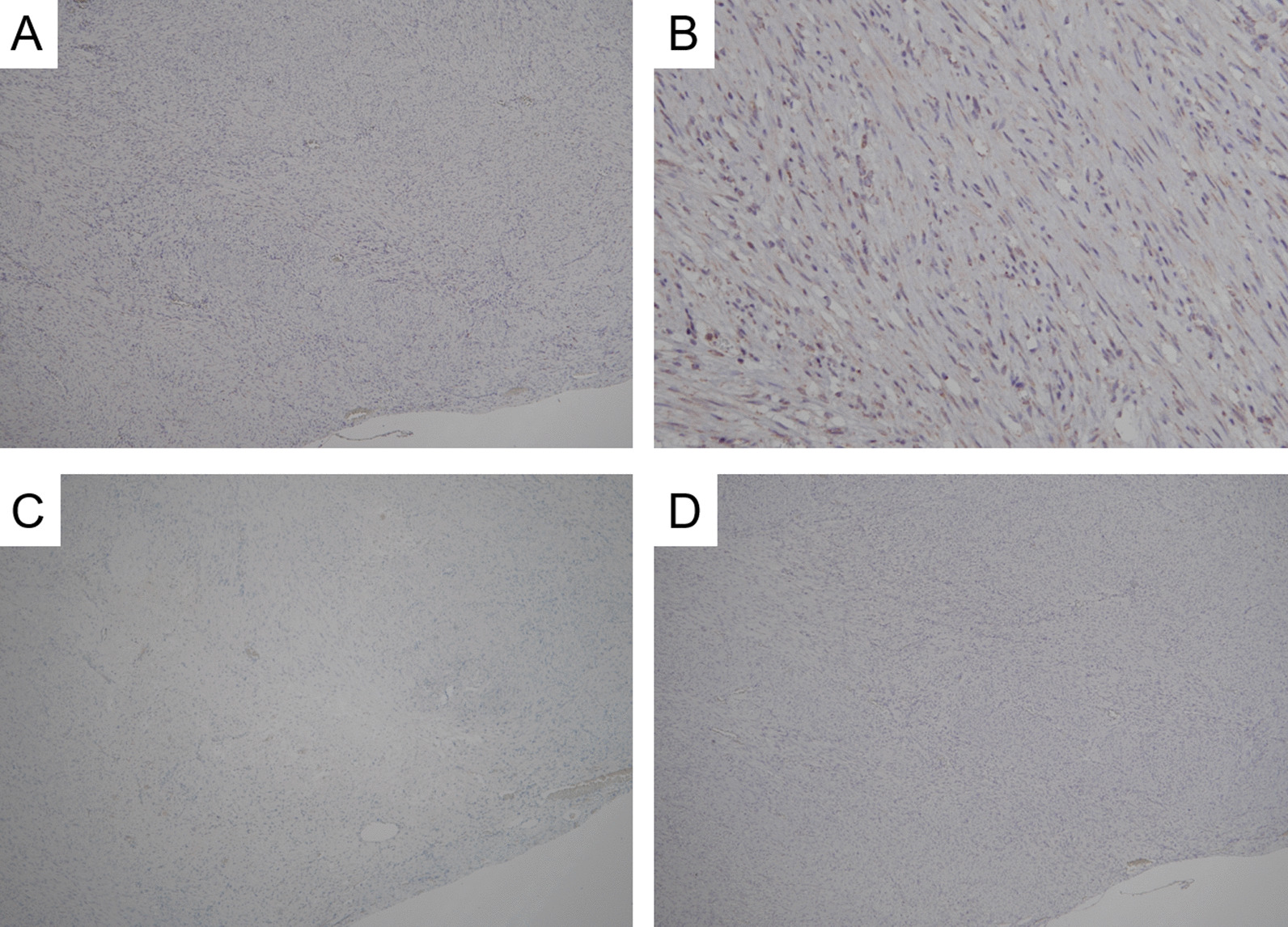
Representative images showing no immunoreactivity in this tumor. **A**–**D** Tumor cells show no immunoreactivity to c-kit, STAT6, HMB45, and CDK4. STAT6 is faintly positive in the cytoplasm but not in the nucleus and is, thus, determined to be negative (**A** c-kit, **B** STAT6, **C** HMB45, **D** CDK4; magnification for **A**, **C**, and **D** ×100 and for **B** ×200)

On immunohistochemical examination, tumor cells showed positive immunoreactivity focally to αSMA and diffusely to HHF35 and desmin (Fig. 4). The Ki-67 labeling index was 1.76% (261/14806) using the average method, while it was 3.51% (43/1226) using the hotspot method [8, 9]. We counted the Ki-67 labeling index using the “Patholoscope” analysis software (MITANI Corporation, Japan, URL: http://www.mitani-visual.jp/en/products/bio_imaging_analysis/patholoscope/), as previously described [10, 11]. Meanwhile, the tumor cells showed negative immunoreactivity for CD34, c-kit, DOG-1, STAT6, S100, HMB45, Melan A, CDK4, MDM2, β-catenin, calretinin, WT-1, estrogen receptor, and progesterone receptor (Fig. 5). In addition, Epstein–Barr virus-encoded RNA in situ hybridization (EBER ISH) showed negative signals in all tumor cells.

Immunohistochemical examination was performed, and no findings suggestive of malignancy (e.g., irregular nuclear shape, mitotic figures, and tumor necrosis) were noted. The results showed smooth muscle marker expression (positive immunoreactivity for αSMA, HHF35, and desmin). In addition, no findings indicating other histological types were found; thus, leiomyoma was considered. However, primary omentum leiomyomas have rarely been reported, except for parasitic leiomyomas [3, 12]. It is also sometimes difficult to completely rule out the malignant potential in smooth muscle tumors in deep soft tissue [13, 14], even in tumors without nuclear atypia, mitotic figures, and coagulopathic tumor necrosis [15].

Therefore, the patient was finally diagnosed with a primary omental smooth muscle tumor considering leiomyoma. The patient consequently did not undergo additional adjuvant therapy and was followed up. Neither recurrence nor metastasis was found on the 13-month postoperative follow-up.

## Discussion and conclusion

We report the case of a primary omental smooth muscle tumor that histologically showed no definite malignant findings in an adult male patient. Except for parasitic leiomyoma, primary omental smooth muscle tumors [16] are extremely rare, and only a few cases have been reported [3, 12]. Tumors arising from the deep soft tissue mainly occur in middle-aged adults with no sex predilection [15]. Histological assessment using imaging is typically difficult, and detailed histological analysis is important for diagnosis [15, 17, 18]. The diagnostic criteria for primary leiomyomas from deep soft tissues are stringent [14]. The diagnosis should be made only after compliance with the following criteria: no nuclear atypia, no or few mitotic figures, and no coagulopathic tumor necrosis on the whole specimen [14].

However, it is difficult to completely rule out the malignant potential in deep soft tissue smooth muscle tumors, even in cases that meet these criteria, and the possibility of a definitive diagnosis of leiomyoma remains controversial [19]. The Ki-67 labeling index is widely known as an indicator of proliferative activity of tumors [20–23], and the average is reported to be 0.52 ± 1.32% [mean ± standard deviation (SD)] in extrauterine leiomyomas [24]. The Ki-67 labeling index using the average method of the current case is within the mean ± SD range of a previous study, but it is close to the upper limit of the mean value plus one SD of the value [24]. Moreover, the Ki-67 labeling index using the hot spot method is 3.5%, which exceeds the mean value plus one SD. If the value is significantly high, leiomyosarcoma can be considered. Nevertheless, if the value is questionable, there are no clear criteria for interpreting the Ki-67 labeling index of smooth muscle tumors arising from deep soft tissue. Therefore, the validity of the hotspot method, the number of tumor cells counted, and the appropriate method for determining the cutoff value remain controversial.

The hotspot method, widely applied to neuroendocrine tumors, might be reasonable [25, 26]. Further analysis is required to define the number of cells counted and cutoff values. There are also smooth muscle tumors of uncertain malignant potential [14, 15, 27, 28]. However, it is unclear whether it can be considered in all cases arising from deep soft tissue, even in cases with no findings indicating malignancy. Therefore, further case reports with long-term follow-up and case series are required to determine whether a true omental benign smooth muscle tumor (leiomyoma) exists. While the current patient was male and we did not necessarily consider a parasitic leiomyoma (an ectopic leiomyoma that arises separately from the uterus), parasitic leiomyoma should be considered in female patients [29]. It is important to confirm the absence of a history of laparoscopic leiomyomectomy or hysterectomy [30]. In some cases, Epstein–Barr virus (EBV)-associated smooth muscle tumor is also a differential diagnosis. In the present case, EBER ISH showed negative signals [31].

We also shed some light on the differential diagnosis from the perspective of spindle cell tumors with relatively little atypia, considering the omental primary. The following types of tumors should be considered: gastrointestinal stromal tumor (GIST), solitary fibrous tumor (SFT), schwannoma, perivascular epithelioid cell tumor (PEComa), and a sclerosing variant of well-differentiated liposarcoma. Extra-GISTs are rare, but several cases have been reported [32–36]. Tumors in the greater omentum are frequently diagnosed as GIST [34]. Immunostaining for CD34 and c-kit can be helpful, but because approximately 5% of the cases show negative results, positivity for other GIST marker expressions, including DOG-1, should also be confirmed [37].

Notably, even GIST rarely shows immunoreactivity to desmin; therefore, other smooth muscle markers should also be evaluated [38]. The presence of a patternless pattern and CD34 immunoreactivity are traditionally common in SFT [39], and the immunoreactivity of STAT6 has recently been emphasized [40, 41]. In addition, smooth muscle markers are negative [42], which is a point of differentiation. Schwannomas often have a morphological mixture of high cell density (Antoni type A) and low cell density (Antoni type B) [43]. Tumor cells show regular- and spindle-shaped nuclei with wavy cytoplasms. Typically, this tumor shows diffuse S100 immunoreactivity and can be differentiated by its negative immunoreactivity for smooth muscle markers [44]. PEComa consists of a mixture of spindle smooth muscle tissue, as well as various types of blood vessels and adipose tissue. However, the proportion of these cells varies among cases, and wholly spindle-shaped tumor cells have been reported [45]. Therefore, it is important to confirm the immunoreactivity of markers, such as HMB45 and Melan A [28, 46].

The sclerosing variant of well-differentiated liposarcoma is extremely rare, but it is a morphological differential disease owing to the lack of fatty components and nuclear atypia [47]. The presence of typical lipoblasts or atypical stromal cells in the surrounding adipose tissue, confirmation of markers, such as CDK4, MDM2, and p16 [48], and negative smooth muscle marker expression are the distinguishing characteristics. Furthermore, confirmation of MDM2 gene amplification by fluorescent *in situ* hybridization is helpful if immunostaining is unsuccessful [49].

We encountered an extremely rare case of primary smooth muscle tumor of the greater omentum in an adult male patient with no histological findings suggestive of malignancy. However, omental smooth muscle tumors are extremely difficult to define as benign; therefore, further case reports with long-term follow-up and case series are required in the future to determine whether a true omental benign smooth muscle tumor (leiomyoma) exists. In addition, proper interpretation of the Ki-67 labeling index should be established (i.e., the validity of the hotspot method, the number of tumor cells to be counted, and the appropriate method to determine the cutoff value). We report this case to emphasize that this tumor requires careful diagnosis and hope that this will act as a foundation for future research.

## Data Availability

The dataset supporting the conclusions of this study is included within the article, and all materials are available upon reasonable request.

## Abbreviations

OALT: Omentum-associated lymphoid tissues
CT: Computed tomography
EBER ISH: Epstein–Barr virus-encoded RNA in situ hybridization
SD: Standard deviation
GIST: Gastrointestinal stromal tumor
SFT: Solitary fibrous tumor
PEComa: Perivascular epithelioid cell tumor
